# cDNA Cloning and Expression Pattern of Homolog of Alpha Subunit of Platelet-Activating Factor Acetylhydrolase Ib from the Chinese Oak Silkworm, *Antheraea pernyi*

**DOI:** 10.1673/031.011.14801

**Published:** 2011-11-08

**Authors:** Yu-Ping Li, Yan-Qun Liu, Huan Wang, Run-Xi Xia, Sheng-Lin Shi, Xian Liu, Shi-Fu Wang, Li Qin

**Affiliations:** Department of Sericulture, School of Life Sciences, Shenyang Agricultural University, Shenyang 110866, China

## Abstract

Platelet-activating factor acetylhydrolase (PAF-AH) is an enzyme that catalyzes the hydrolysis of platelet-activating factor (PAF). A homolog of alpha subunit of PAF-AH(Ib) from *Antheraea pernyi* (Guérin-Méneville) (Lepidoptera: Saturniidae) (*ApPAFAHIbα*) was isolated and characterized. The obtained cDNA sequence was 1843 base pairs (bp) long with an open reading frame (ORE) of 678 bp encoding 225 amino acids. The predicted amino acid sequence shared several conserved features of PAF-AHs of other organisms, and revealed 88, 60, and 46% identity with the homologues of *Bombyx mori, Drosophila melanogaster*, and *Homo sapiens*, respectively. Phylogenetic analysis indicated that lepidopteran PAFAHIbαs including ApPAFAHIbα might be a new member of the PAF-AHs family of insects. Reverse transcriptase polymerase chain reaction (RT-PCR) analysis showed that the *ApPAFAHIbα* gene was transcribed at four developmental stages and expressed in all tissues tested.

## Introduction

Platelet-activating factor acetylhydrolase (PAF-AH; EC 3.1.1.47) is an important enzyme that catalyzes the hydrolysis of platelet-activating factor (PAF). PAF (1-*O-*alky1-2-acety1-*sn*-glycero-3-phosphocholine) is one of the most potent lipid mediators and is involved in a variety of physiological events (Peplow 1999). The acetyl group at the *sn*-2 position of the glycerol backbone is required for its biological activity, and deacetylation of PAF induces loss of activity (Stafforini 2009). PAF-AH is a calcium independent phospholipase A2 that exhibits strong substrate specificity towards PAF, hydrolyzing an acetyl ester at the *sn*-2 position. It serves an anti-inflammatory function by converting the proinflammatory autocoid, PAF, into biologically inactive lyso-PAF by the removal of the *sn*-2 acetyl group of this glycerophospholipid. Similarly, PAFAHs can also degrade oxidatively modified *sn*-2 polyunsaturated-fatty-acid-containing phospholipids, which are toxic to cells. In addition, PAF and PAF-AH are associated with neural migration and mammalian reproduction. Therefore, PAF-AH is a defense mechanism that protects the host against the toxic effects of PAF and other biologically active oxidized phospholopids (Lee et al. 2005).

Three types of PAF-AH have been identified in mammals, namely the intracellular type Ib and II and plasma type (Karasawa et al. 2003; Chen 2004). These enzymes show different biochemical properties and molecular structures. According to the specificity and the chemical nature of the substrate molecules, PAF-AH has been classified as a group VII phospholipase A_2_ (Tjoelker et al. 1995; Stafforini et al. 1997). Two homologs of the alpha and beta subunits of PAF-AH(Ib) have been observed in fruit fly *Drosophila melanogaster* (Sheffield et al. 2000). As already pointed out, the PAF-AH(Ib) is an oligomeric complex, and its physiological function is not yet understood (Sheffield et al. 2000).

The Chinese oak silkworm, *Antheraea pernyi* (Guérin-Méne ville) (Lepidoptera: Saturniidae), is one of the most well known wild silkmoths used for silk production. Recently, it has mainly been used as a source of insect food. This insect is known to have been domesticated in China around the 16th century (Liu et al. 2010a), and is commercially cultivated today in China, India, and Korea. To identify more *A. pernyi* genes, we have constructed a full-length cDNA library from *A. pernyi* pupa (Li et al. 2009). By cDNA library screening, several *A. pernyi* genes encoding important enzymes have been cloned and characterized, such as two *enolase* genes (Liu et al. 2010b) and a *lysophospholipase* gene (Liu et al. 2010b).

This work describes the cloning and characterization of the homolog of alpha subunit of PAF-AH(Ib) from *A. pernyi* pupal cDNA library, which was named as ApPAFAHIb*α*. The deduced protein sequence was compared to other organisms and the expression patterns at various developmental stages and in different tissues of fifth instar larvae were determined. The results presented here provide the basic information for its functional analysis.

## Materials and Methods

### Silkworms and tissues

The *A. pernyi* strain *Shenhuang No. 1* was used in this study. Larvae were reared routinely on oak trees, *Quercus liaotungensis* Koidz (Fagales: Fagaceae), in the field. Blood, fat body, midgut, silk glands, body wall, Malpighian tubules, spermaries, ovaries, brain and muscle were taken from silkworm larvae at day 10 of fifth instar and immediately frozen in liquid nitrogen and stored at -80° C. Eggs at day 5, larvae of fifth instar, pupae, and moths were also stored at — 80° C for later use.

### Cloning of the *ApPAFAHIb-α* gene and sequence analysis

A full-length cDNA library of *A. pernyi* pupa has been constructed (Li et al. 2009). An EST encoding PAFAHIbα homolog (GenBank accession no. GH335042) was isolated by random EST sequencing. The cDNA clone was used to complete the full-length cDNA sequence of the *ApPAFAHIb-α* gene. DNASTAR software (DNASTAR Inc., www.dnastar.com) was used to identify open reading frame (ORF), deduce amino acid sequence, and predict the isoelectric point and molecular weight of the deduced amino acid sequence. Blast search was performed at www.ncbi.nlm.nih.gov/blast/. The deduced amino acid sequence was submitted to predict protein signal peptide with SignalIP server online tool (www.cbs.dtu.dk/services/SignalP/). Prediction of Subcellular Localization was performed at www.bioinfo.tsinghua.edu.cn/SubLoc/. Transmembrane protein topological structure was analyzed with TMHMM server on-line tool (www.cbs.dtu.dk/services/TMHMM/). Conserved Domains was predicted at www.ncbi.nlm.nih.gov/Structure/cdd/wrpsb.cgi/. The *in silico* gene expression analysis based on the available EST resources was employed at www.ncbi.nlm.nih.gov/Unigen/ESTprofileViewer/.

### Total RNA extraction and first strand cDNA synthesis

Total RNA was extracted by using RNAsimple Total RNA Extraction Kit (Tiangen Biotech, www.tiangen.com) according to manufacturer instructions. The purity and quantity of the extracted RNA was quantified by the ratio of OD_260_/OD_280_ by ultraviolet spectrometer. First strand cDNA was generated by using 2 µg of total RNA per sample with TIANScript cDNA Synthesize Kit (Tiangen Biotech, www.tiangen.com).

### RT-PCR analyses

The cDNA samples were amplified by the semi-quantitative polymerase chain reaction (PCR) method using the gene-specific primer pair LYQ120 (5′ TGGTT TGCTC CACTT CACTG 3′) and LYQ121 (5′ CTTTT TCTGG TTCAC CCTCA 3′) for the *ApPAFAHIbα* gene, which generated a 490 base pair (bp) fragment. An *actin* gene (GU073316) was used as an internal control, and a 468 bp fragment was amplified in parallel to each RNA sample using the primer pair LYQ85 (5′ CCAAA GGCCA ACAGA GAGAA GA 3′) and LYQ86 (5′ CAAGA ATGAG GGCTG GAAGA GA 3′) (Wu et al. 2010). PCRs were performed with the following cycles: initial denaturation at 95° C for five minutes followed by 30 cycles of one minute at 95° C, 30 seconds annealing at 55° C, 30 seconds extension at 72° C, and a final extension at 72° C for 10 minutes. The amplification products were analyzed on 1.0% agarose gels, purified from the gel, and directly sequenced.

### Phylogenetic analysis

The amino acid sequences of PAFAHIb*α* homologs from different organisms were retrieved from GenBank database. Multiple sequence alignments were performed using Clustal X software (Thompson et al. 1997). A phylogenetic tree was constructed by MEGA version 4.0 (Tamura et al. 2007) using the Neighbor-Joining (NJ) method (Saitou and Nei 1987) with bootstrap test of 500 replications.

**Figure 1.**
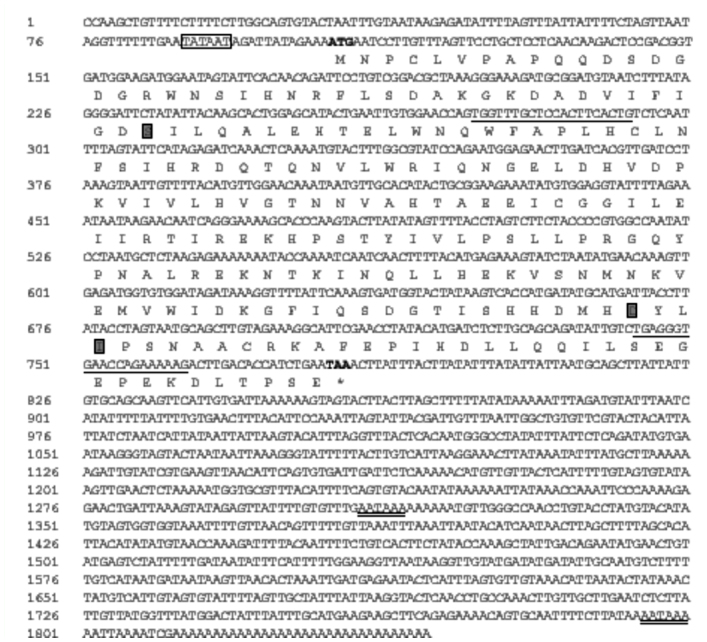
The complete nucleotide and deduced amino acid sequence of the homolog of alpha subunit of PAF-AH(Ib) of *Antheraea pernyi*. The amino acid residues are represented by one-letter symbols. The initiation codon ATG is indicated with bold and termination codon TAA is indicated with bold and by an asterisk. The TATA box is boxed. Predicted catalytic triad is boxed in grey squares. The polyadenylation signal AATAAA is double-underlined. The underlined nucleotides show the positions of gene specific primers used in the experiment. The cDNA sequence was deposited in GenBank under accession no. GU28992S. High quality figures are available online

## Results

### cDNA cloning of the *ApPAFAHIbα* gene

The *ApPAFAHIbα* gene was identified from the *A. pernyi* pupal cDNA library. Based on the EST clone Appu0212, a full-length cDNA clone of the *A. pernyi* PAF-AH(Ib) alpha subunit homolog was isolated and sequenced. The cDNA sequence and deduced amino acid sequence of the *ApPAFAHIbα* gene are shown in Figure 1. The obtained 1843 bp cDNA sequence contains a 5′-untranslated region (UTR) of 105 bp with one TATA box (5′TATAAT), a 3′ UTR of 1028 bp with a polyadenylation signal sequence AATAAA at position 1795, a poly (A) tail, and an ORF of 678 bp encoding a polypeptide of 225 amino acids. However, another possible polyadenylation signal sequence is present at position 1059 of the cDNA. The ApPAFAHIbα protein has a predicted molecular weight of 25.60 kDa and isolectric point of 5.7. Blast search revealed that the deduced amino acid sequence of the *ApPAFAHIbα* gene had 88% identities and 95% positives with that of the putative *Bombyx mori* PAFAH(Ib) alpha subunit homolog (ABF51262). Conserved Domains prediction showed that it contained the PAFAH domain with several conserved features, such as the catalytic triad Ser^43^-Asp^188^-Lle^191^ in the active sites which resembles the typical Ser-Asp(Glu)-His catalytic triad (Sheffield et al. 2000), the oxyanion hole Ser^43^-Arg^70^-Asn^100^, and the specificity pocket Ile^44^-Thr^99^Leu^190^. This cDNA sequence has been deposited in GenBank under accession no. GU289925.

Prediction of subcellular localization indicated that this protein is a cytoplasmic protein (Reliability Index: RI = 1; Expected Accurcy = 56%). Protein signal peptide prediction revealed no deduced signal peptide cleavage site in the N-terminal (Signal peptide probability: 0.000; Signal anchor probability: 0.000; Max cleavage site probability: 0.000 between positions 15 and 16), indicating a non-secretory protein. No transmembrane helices were detected in this protein by transmembrane protein topological structure analysis.

### Homologous alignment and phylogenetic analysis

**Figure 2.**
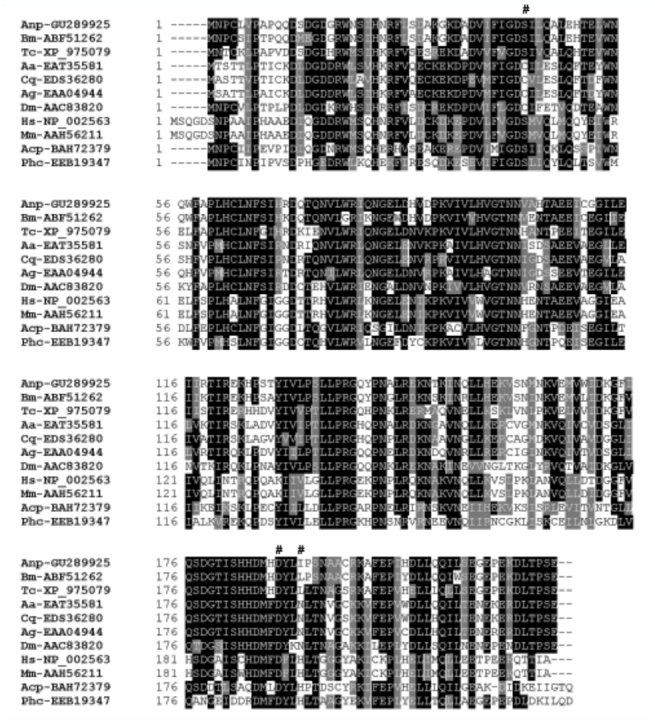
Sequence alignment of PAF-AH(Ib) proteins from *Antheraea pernyi* and other organisms. The number sign (#) show the residues in which the mammalian proteins form the catalytic triad. GenBank accession numbers of PAF-AH proteins are shown following the names of organisms. Anp, *Antheraea pernyi*; Bm, *Bombyx mori*; Tc, *Tribolium castaneum*; Aa, *Aedes aegypti*; Cq, *Culex quinquefasciatus*; Ag, *Anopheles gambiae*; Dm, *Drosophila melanogaster*; Acp, *Acyrthosiphon pisum*; Phc, *Pediculus humanus corporis*; Hs, *Homo sapiens*; Mm, *Mus musculus*. High quality figures are available online

**Figure 3.**
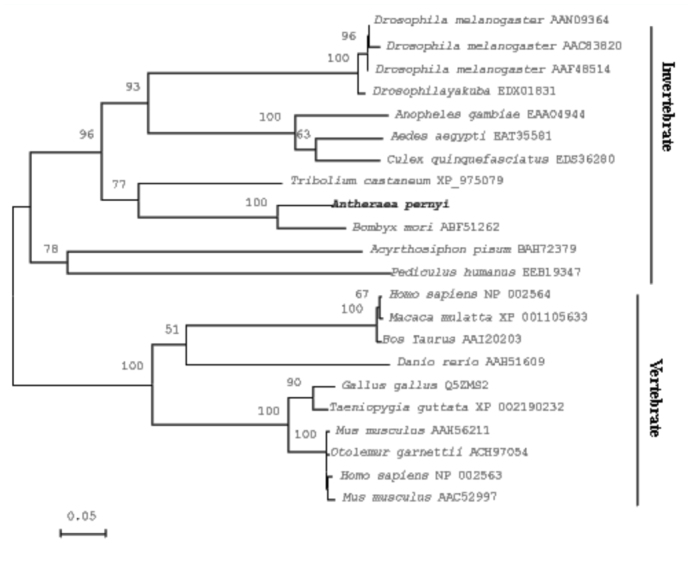
Phylogenetic tree based on the amino acid sequence comparisons of PAF-AH(Ib) proteins from various organisms made with MEGA 4.1 software using Neighbor-Joining (NJ) method. The numbers above the branch represent bootstrap percentages. The topology was tested using bootstrap analyses (500 replicates). GenBank accession numbers of PAF-AH proteins are shown following the names of the insect. High quality figures are available online

To assess the relatedness of ApPAFAHIb*α* to PAF-AH(Ib) proteins from other organisms, identities were calculated based on a Clustal alignment including 22 PAF-AH(Ib) protein sequences (Figures 2 and 3). Sequence alignment revealed that the length of the coding region of the *ApPAFAHIbα* gene compared with those of homologs from other organisms was highly conserved. However, the ApPAFAHIbα protein revealed 88, 71, 60, 50, 47, and 46% identity with the homologs of *B. mori* (ABF51262), *Tribolium castaneum* (XP_97579), *Drosophila melanogaster* (AAN09364), *Acyrthosiphon* pisum (BAH72379), *Mus musculus* (AAH56211), and *Homo sapiens* (NP_002563), respectively (Figure 2). The results showed that there is an extremely high degree of sequence divergence among these PAF-AH(Ib) proteins, suggesting that the *PAF-AH(Ib)* genes have had a long independent evolutionary history as they have come from phylogenetically distant organisms.

The phylogenetic tree constructed by MEGA version 4.0 (Tamura et al. 2007) using the NJ method (Saitou and Nei 1987) is shown in Figure 3. The PAF-AH(Ib) sequences were well divided into two groups corresponding to invertebrate and vertebrate. Among insects, the alpha subunit of ApPAF-AH(Ib) has a closer relationship to the homologs in *B. mori* followed by *T. castaneum*. The results agreed with morphological classification and other molecular data such as the *lysophospholipase* gene (Liu et al. 2010a) and *enolase* gene (Liu et al. 2010b). The two PAF-AH(Ib) proteins from Lepidopterans were grouped into a novel cluster in phylogenetic tree, indicating that the lepidopteran PAF-AH(Ib)S might be a new member of insect PAF-AH(Ib) proteins.

### Expression patterns at different stages and in different tissues

**Figure 4.**
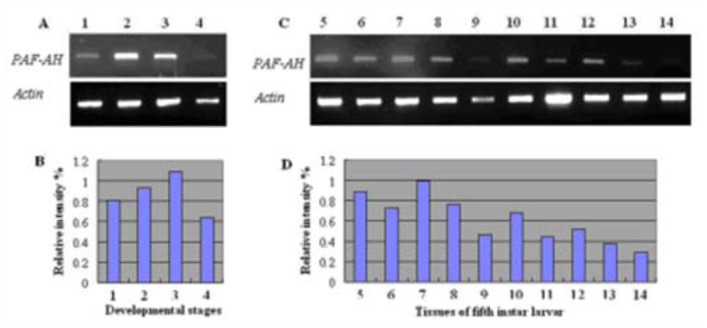
Expression patterns *of ApPAFAHIbα* mRNA in different developmental stages (A and B) and different tissues of fifth instar larvae (C and D) were performed by semi-quantitative RT-PCR. RT-PCR was amplified after 30 cycles with a specific primer pair for the *ApPAFAHIbα* gene. The *actin* gene was used as an internal standard to normalize the templates. Relative expression profiles *of ApPAFAHIbα* were normalized with *actin* level. Lanes: I, eggs at day 5; 2, larvae of fifth instar; 3, pupae; 4, moths; 5, blood; 6, fat body; 7, midgut; 8, silk glands; 9, body wall; 10, Malpighian tublues; 11, spermaries; 12, ovaries; 13, brain; 14, muscle. High quality figures are available online

Semi-quantitative RT-PCR was performed to detect and quantify the *ApPAFAHIbα* gene expression levels during different developmental stages and tissue distributions in fifth instar larvae by using an *actin* gene as an internal control that was constitutively expressed (Wu et al. 2010). The results showed that the *ApPAFAHIbα* gene was expressed during four developmental stages; egg, larva, pupa, and adult (Figure 4A). This was consistent with the results of *in silico* gene expression of alpha subunit homologs from *B. mori* and *D. melanogaster* based on the available EST resources. The *in silico* gene expression analysis showed that *B. mori* alpha subunit homolog was expressed during four developmental stages. Analysis of *in silico* gene expression showed that *D. melanogaster* alpha subunit homolog was also expressed during four developmental stages, consistent with the observations by Northern blots (Sheffield et al. 2000). The expression levels of *ApPAFAHIbα* in the pupal stage were highest among the four developmental stages tested (Figure 4B). These results suggested that the product of the homolog of PAF-AH(Ib) alpha subunit plays an essential role throughout the entire life cycle of insect.

Tissue distributions of the *ApPAFAHIbα* gene in fifth instar larvae were also analyzed. The results showed that *ApPAFAHIbα* RNA was present in all tissues tested including blood, fat body, midgut, silk glands, body wall, Malpighian tubules, spermaries, ovaries, brain, and muscle (Figure 4C). The mRNA levels were most abundant in midgut and blood, contrasting with much lower levels in the brain and muscle (Figure 4D). The *in silico* gene expression analysis based on the available EST resources showed that the *B. mori* alpha subunit homolog was expressed in silk glands, ovaries, and spermaries, and that the *D. melanogaster* alpha subunit homolog was found in fat body and ovaries. However, analysis of *in silico* gene expression based on the available EST resources showed that human PAF-AH(Ib) alpha subunit was ubiquitously expressed in almost all tissues.

## Discussion

In the present study, the homolog of the alpha subunit of the PAF-AH(Ib) from *A. pernyi* was cloned by screening the pupal cDNA library. To date, there are 19 sequences of insects in the GenBank database for the homologs of the PAF-AH(Ib) alpha subunit including *Drosophila, Aedes aegypti, Anopheles gambiae, Culex quinquefasciatus, Pediculus humanus, A. pisum, T. castaneum*, and *B. mori*. There are, however, no reports in the literature of homologs of this protein other than that of the fruit fly (Sheffield et al. 2000). This is the second report of the cloning and characterization of the gene encoding the alpha subunit of the PAF-AH(Ib) of any insect species. The extremely high degree of divergence in amino acid sequences of PAFAH(Ib) proteins in insects suggests that PAFAH(Ib) homologs have had a long independent evolutionary history. On the other hand, the level of the amino acid conservation of the alpha subunit of PAFAH(Ib) from insect to human is remarkably high at more than 46% identity, but is consistent with the reported strict conservation of these proteins within mammals. The high level of conservation is suggestive of a critical function that these proteins must play in all organisms where they are found (Sheffield et al. 2000).

By sequence alignment it was determined that within mammals including *H. sapiens* (NP_002563 and NP_002564), *M. musculus* (AAH56211 and AAC52997), *Bos Taurus* (AAI20203), *Danio rerio* (AAH51609), *Gallus gallus* (Q5ZMS2), *Macaca mulatta* (XP_001105633), *Otolemur garnettii* (ACH97054), and *Taeniopygia guttata* (XP_002190232), the active site residues of the homologs of alpha subunit of PAFAH(Ib), which form the catalytic triad Ser^48^Asp^193^-His^196^ (human PAF-AH(Ib) with accession no. NP_002563) are identical (Figure 2 and data not shown). However, in spite of the significant sequence similarity between insect PAF-AH(Ib) and these mammalian homologs, the active site residues forming the catalytic triad Ser-Asp-His are not conserved. The nucleophilic Ser^48^ of human PAF-AH(Ib) is replaced by a cysteine within the dipterans *Drosophila, A. aegypti, A. gambiae,* and *C. quinquefasciatus*, whereas it does not change among the other insects including *A. pernyi*. The Asp^193^ is conserved in all homologs available to date. The His^196^ of the catalytic triad is identical in *P. humanus* and *A. pisum*, whereas it is replaced by an asparagine in dipterans, a leucine in *T. castaneum* and *B. mori*, and an isoleucine in *A. pernyi*. It has been shown that the *D. melanogaster* homolog lacks catalytic activity as two of the three residues in the putative catalytic triad are missing (Sheffield et al. 2000). The conclusion has been made that the protein of *Drosophila* homolog plays a non-catalytic role and that mammalian protein may also have a primary physiological function that is not catalytic. It will be interesting to see if the *A. pernyi* protein is sufficient to display catalytic activity. The overexpression, purification, and PAF-AH catalytic activity of the *A. pernyi* protein are currently under way. In summary, the full-length cDNA encoding a homolog of alpha subunit of PAF-AH(Ib) from *Antheraea pernyi* has been cloned and characterized. It was shown that the *A. pernyi* PAF-AH(Ib) alpha subunit homolog was transcribed at four developmental stages and ubiquitously expressed in all tissues tested, and that the blood and midgut are the main PAF-AH(Ib) producing tissues. The results demonstrate that the active site residues forming the catalytic triad Ser-Asp-His are not conserved despite the significant sequence similarity between insect PAF-AH(Ib) alpha subunit homologs and these mammalian homologs. Future research should focus on determining the catalytic activity of the *A. pernyi* protein.
